# Probing Interactions between AuNPs/AgNPs and Giant Unilamellar Vesicles (GUVs) Using Hyperspectral Dark-field Microscopy

**DOI:** 10.3390/ijms19041014

**Published:** 2018-03-28

**Authors:** Anupama Bhat, Kewei Huan, Tiana Cooks, Hacene Boukari, Qi Lu

**Affiliations:** 1 Department of Physics and Engineering, Delaware State University, 1200 N. DuPont Hwy, Dover, DE 19977, USA; abhat13@students.desu.edu (A.B.); tdcooks15@students.desu.edu (T.C.); hboukari@desu.edu (H.B.); 2 School of Science, Changchun University of Science and Technology, 7089 Weixing Rd, Changchun 130022, China; huankewei@126.com

**Keywords:** gold nanoparticles, silver nanoparticles, giant unilamellar vesicles, dark-field microscopy, hyperspectral imaging, DMPC, cholesterol

## Abstract

Noble metallic nanoparticles (NPs) such as gold and silver nanoparticles (AuNPs and AgNPs) have been shown to exhibit anti-tumor effect in anti-angiogenesis, photothermal and radio therapeutics. On the other hand, cell membranes are critical locales for specific targeting of cancerous cells. Therefore, NP-membrane interactions need be studied at molecular level to help better understand the underlying physicochemical mechanisms for future applications in cancer nanotechnology. Herein, we report our study on the interactions between citrate stabilized colloidal AuNPs/AgNPs (10 nm in size) and giant unilamellar vesicles (GUVs) using hyperspectral dark-field microscopy. GUVs are large model vesicle systems well established for the study of membrane dynamics. GUVs used in this study were prepared with dimyristoyl phosphatidylcholine (DMPC) and doped with cholesterol at various molar concentrations. Both imaging and spectral results support that AuNPs and AgNPs interact very differently with GUVs, i.e., AuNPs tend to integrate in between the lipid bilayer and form a uniform golden-brown crust on vesicles, whereas AgNPs are bejeweled on the vesicle surface as isolated particles or clusters with much varied configurations. The more disruptive capability of AuNPs is hypothesized to be responsible for the formation of golden brown crusts in AuNP-GUV interaction. GUVs of 20 mol% CHOL:DMPC were found to be a most economical concentration for GUVs to achieve the best integrity and the least permeability, consistent with the finding from other phase studies of lipid mixture that the liquid-ordered domains have the largest area fraction of the entire membrane at around 20 mol% of cholesterol.

## 1. Introduction

Noble metallic nanoparticles (NPs) such as gold and silver nanoparticles (AuNPs and AgNPs) are clusters of tens to thousands of gold or silver atoms with sizes ranging from 1 to 100 nm. They have been increasingly integrated into a wide array of biomedical applications owing to their unique optoelectronic characteristics and surface chemistry as well as the possibility of well-controlled synthesis. Being biocompatible and inert [1,2], AuNPs are very attractive for biomedical and pharmaceutical applications [3,4], such as drug and gene delivery [5], medical diagnostics [6,7], and therapeutics [8,9]. On the other hand, silver nanoparticles (AgNPs) have long been known for their potent antimicrobial and anti-inflammatory effects for such applications as wound dressing and biomedical implants [10]. Both gold and silver NPs have been shown to exhibit anti-tumor effect through inhibiting the inherent function of heparin binding growth factors thereby suppressing the abnormal growth of blood vessels [11]. Multiple animal studies have shown that gold or silver NPs can significantly reduce the tumor size and enhance the survival rate of animals in either photothermal or radio therapy [11]. Given the promise of gold or silver NPs in targeted elimination of cancerous cells while sparing the normal tissue [11,12,13], more research is needed at molecular levels for the development of safer, robust, effective and efficient gold and silver NP-based therapeutic systems.

On the other hand, cell membranes are critical locales for the specific targeting of cancerous cells, yet the fundamental mechanisms that govern the interactions of gold or silver NPs and cell membranes remain largely inconclusive. The inevitable adsorption of nanomaterials on the surface of membranes modifies the physicochemical properties of the membrane [14]. Therefore, it is imperative to conduct studies based on simplified model systems for the physicochemical factors to be revealed on interactions occurring between nanoparticles and biological membranes.

Giant unilamellar vesicles (GUVs) are the model membrane system used in this study, because they provide a cell-sized confined volume for the study of biochemical reactions as well as self-assembly processes that allow for tunable lipid composition. The size of GUVs ranges from 1–100 µm, making them easily distinguishable using light and fluorescence microscopy. The simplest GUV consists of a spherical lipid bilayer enclosing a buffer. This membrane bound entity mimics three important features of a cell, namely: compartmentalization, finite surface area and bending elasticity of the cellular membranes. GUVs allow optical examinations at the single-vesicle level thus offer a facile model system to study fundamental membrane thermodynamics, membrane domains and mechanical properties such as membrane curvature, membrane morphology and shape changes [15]. Since phosphatidylcholine is the most abundant lipid in the membranes of eukaryotic cells, the base composition of the GUVs used in this study was chosen to be dimyristoyl phosphatidylcholine (DMPC). DMPC is a widely used zwitterionic phospholipid molecule in model membranes with 14 carbon atoms in the alkyl chain and a melting temperature at 24 °C.

The composition of lipid membranes is an important factor which influences the interaction of NPs with the membrane. Cholesterol (CHOL) is a dominant sterol component in mammalian cell membranes that regulates the fluidity of the latter. Cholesterol behaves as a spacer molecule or dynamic “glue” which fills in between sphingolipids and phospholipids that enables the tight packing to form the floating membrane microdomains known as lipid rafts [16,17]. Lipid rafts are dynamic liquid-ordered platforms that can include or exclude proteins to various extents [18], thus providing anchorage for receptors, coupling factors, effector enzymes, and substrates for the orchestrated function of cell signaling [17]. The modulation of malignant phenotype of cancer cells in terms of cancer cell adhesion, aggressive invasion and metastatic spread occurs at the surface, to a large extent, mediated by lipid rafts [19]. For example, CD44 is a marker molecule expressed in cancer cells and has been associated with cancer cell adhesion, migration, and metastasis. Its abundance in lipid rafts has been noted in several reports [20,21,22]. Cholesterol depletion treatment has been shown to trigger the shedding of CD44 and hence suppress tumor cell migration [23]. Considering the important role cholesterol plays in regulating the fluidity of cell membranes hence the ability to hold key proteins in cancer therapy, we tested GUVs at various molar concentration of DMPC:CHOL to gain understanding of the effects of cholesterol on GUV-NP interactions.

The major observation technique employed in this work is the dark-field microscopy (DFM), which works by a special optical design that allows only the light scattered from an unstained specimen to be collected by the objective lens while rejecting the illumination. As a result, a brightly-lit image appears against a dark, almost black background with much enhanced signal to noise ratio. Dark-field microscopy is an enabling optical technique for the observation of nanoparticles as small as a few to tens of nanometers under native conditions [24], as the tiny size of nanoparticles is way beyond the resolution limit around 200 nm for regular light microscopes. The other distinctive advantage of DFM, especially in contrast to the fluorescence microscopy, is to enable the observation of biological molecules without conjugation of fluorescence tags. The label-free feature in DMF is advantageous in the investigation of biological systems in a native state, which is of better biomedical relevance as compared to those that are conjugated with fluorescence tags sizable enough to alter the biological condition.

An added benefit of the dark-field microscopy system (CytoViva, Inc., Auburn, AL, USA) used in this work is the formation of hyperspectral imaging (HSI), a technique combining spectrophotometry and imaging [25]. The advanced optics and algorithms built into the system allows for the simultaneous acquisition of the spatial images and the reflectance spectral response at every pixel, nondestructively. Instead of taking a single photograph with only the dominant wavelength, HSI contains the complete spectral response at each pixel which enables the quantitative characterization of NPs and their interactions with biomolecules. The scattering spectra arising from the light-matter interactions encompass implications from the electrical, optical, and plasmonic properties of NPs as well as their local environments. The spectral response can range from the visible near infrared (VNIR) to short wave infrared (SWIR) dependent on the setup of CCD camera. A recent study shows that HSI can enhance the cytologic diagnosis of cancer cells [26].

In this paper, we report our major findings made with hyperspectral dark-field microscopy on the interactions between citrate stabilized colloidal AuNPs or AgNPs and GUVs of varied composition including pure DMPC to different percent molar concentration of CHOL:DMPC (10, 20, 30 and 40 mol%). The size chosen for both AuNPs and AgNPs was 10 nm, because in our earlier work 10-nm AuNPs were found to be most capable in inducing the phase and shape changes in lipid vesicles [27]. The findings reported in this work are intended to provide better understanding regarding the mechanisms of AuNP/AgNP and membrane interactions, which are fundamental and critical for their future applications in both therapeutics and diagnostics of cancer.

## 2. Results

### 2.1. Dark-Field Microscopy (DFM) Images and Spectral Profiles of AuNPs and AgNPs

**Figure 1:**
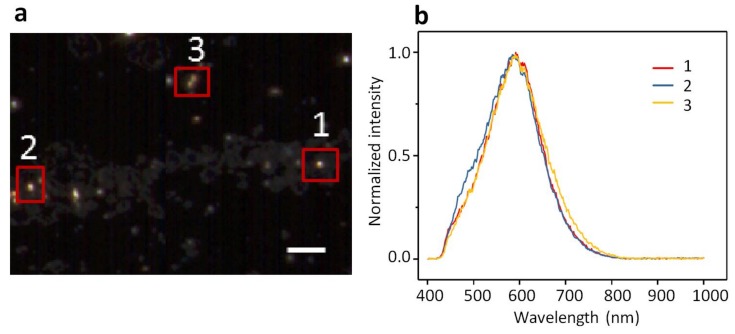
(**a**) A dark-field microscopy (DFM) image of 10-nm AuNPs dispersed in PBS buffer and (**b**) the normalized spectral profiles collected from three different particles specified in (**a**). The three spectral profiles in (**b**) peak are around 590 nm. Scale bar is 5 µm.

**Figure 2:**
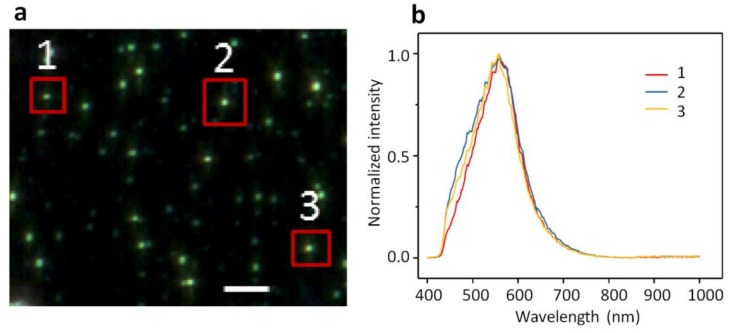
(**a**) A DFM image of 10-nm AgNPs dispersed in PBS buffer and (**b**) the normalized spectral profiles collected from three different particles specified in (**a**). The three spectral profiles in (**b**) peak are around 550 nm. Scale bar is 5 µm.

The dark-field images of AuNPs or AgNPs of 10 nm in size appear as bright and shiny dots against a dark background as shown in Figure 1a and Figure 2a. AuNPs exhibit a yellowish-brown hue whereas AgNPs exhibit a greenish hue. Three representative NPs are highlighted in each image acquired with ENVI hyperspectral imaging. The spectral profiles averaged for each NP are displayed in Figure 1b and Figure 2b, respectively. The spectral profiles from AuNPs peak around 590 nm whereas those from AgNPs peak around 550 nm. The spectral profiles from different NPs vary slightly in shape and width but hold the same peak for both AuNPs and AgNPs (Figure 1b and Figure 2b). The spectral profiles from AuNPs are broader than those from AgNPs. It is also found that the spectral profiles from AuNPs (Figure 1b) are more symmetric than those from AgNPs (Figure 2b), with the spectral profiles from AgNPs leaning slightly toward red.

### 2.2. DFM Images and Spectral Profiles of GUVs Interacting with AuNPs or AgNPs

The GUVs made up of DMPC alone or DMPC doped with varied percent molar concentration of CHOL (10, 20, 30 and 40 mol%) were imaged with or without AuNPs and AgNPs. As found in the analysis of spectral profiles to follow that GUVs of 20 mol% CHOL:DMPC tend to have most prominent shifts in peak wavelength and most broadening in reflectance spectral width, therefore we display the DFM images as well as the spectral profiles obtained from the batch that GUVs were prepared with 20 mol% CHOL:DMPC.

**Figure 3:**
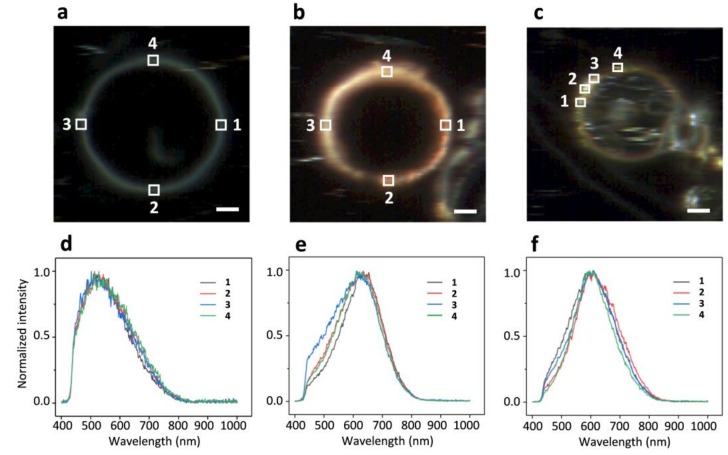
DFM images (**a**) GUV alone, (**b**) AuNPs and GUVs, as well as (**c**) AgNPs and GUVs. The GUVs shown here are made of 20 mol% CHOL:DMPC. (**d**–**f**) are the normalized spectral profiles averaged from inside of the squared regions in images (**a**–**c**), respectively. Scale bars are 5 μm.

Shown in Figure 3 are DFM images for (a) GUVs alone, (b) GUVs with AuNPs and (c) GUVs with AgNPs. The spectral profiles acquired inside the squared regions on each image are shown right below in (d), (e) and (f), respectively. The normalized spectral profiles (Figure 3d) from four different lipid bilayer patch regions of the imaged GUV (Figure 3a, 20 mol% CHOL:DMPC in composition) are identical in shape and all peak around 550 nm. The spectral profiles from GUV+AuNPs (Figure 3e) are broadened and shifted in peak from ~590 nm to ~620 nm as compared to those from AuNPs alone (Figure 1b). Also, the spectral profiles collected at different regions show more variations in shape and width as compared to those from GUV alone (Figure 3d). The variations occur mostly on the left halves of the spectral profiles. The spectral profiles from GUV+AuNPs lose the symmetry as found with AuNPs alone (Figure 1b). Similar spectral broadening is observed for GUV+AgNPs (Figure 3f) as well as the peak shift from ~550 nm to ~590 nm as compared to those from AgNPs alone (Figure 2b). Variations in spectral shape and width are also observed for GUV+AgNPs from different regions. Ironically, spectral profiles from GUV+AgNPs are more symmetric than those from AgNPs alone (Figure 2b).

**Figure 4:**
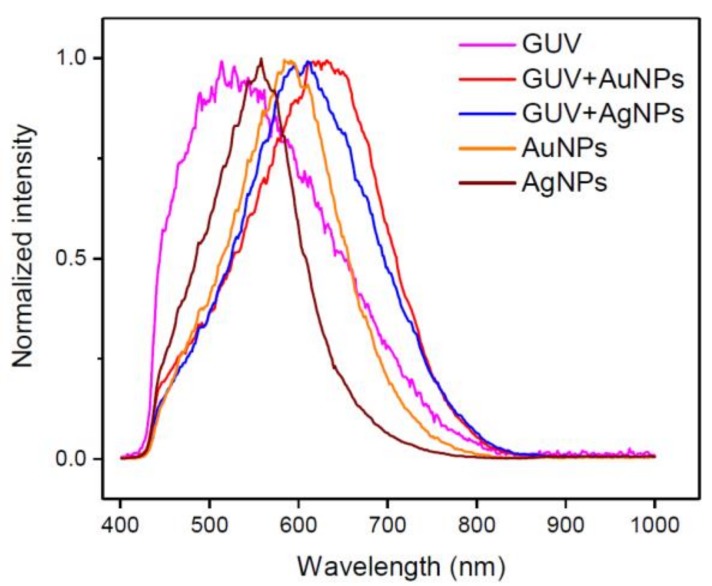
The normalized spectral profiles averaged from the highlighted regions in previous figures for AuNPs, AgNPs, GUVs, GUV+AuNPs, and GUV+AgNPs, respectively. The GUVs shown in this graph were made of 20 mol% CHOL:DMPC.

Shown in Figure 4 are the normalized spectral profiles averaged from the highlighted regions in Figure 1, Figure 2 and Figure 3. Five plots are from AuNPs, AgNPs, GUVs, GUV+AuNPs, and GUV+AgNPs, respectively. The GUVs shown in this graph were made of 20 mol% CHOL:DMPC. The red shifts occur for both AuNPs and AgNPs upon interactions with GUVs. The spectral broadening also occurs for both AuNPs and AgNPs upon interactions with GUVs. The spectral profile of GUV alone is more asymmetric than the other four, leaning toward blue and tapering off toward red. The spectral profiles of AgNPs and GUVs are both asymmetric yet resulting in a symmetric profile upon interactions of AgNPs and GUVs. The spectral profile of AuNPs is relatively symmetric yet resulting in a slightly asymmetric profile upon interacting with GUVs.

**Figure 5:**
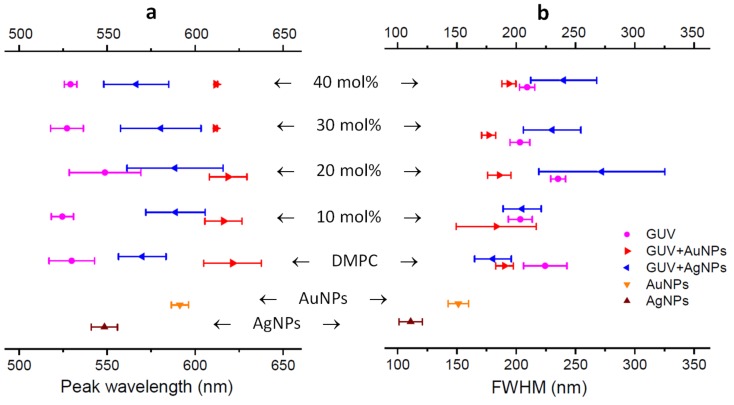
(**a**) The peak wavelengths and (**b**) the FWHM (full width at half maximum) of the spectral profiles from AuNPs alone, AgNPs alone, GUVs alone, GUV+AuNPs, and GUV+AgNPs. The GUV composition was varied from DMPC only to 10, 20, 30 and 40 mol% CHOL:DMPC. The error bars are based on the standard deviations calculated from 15 regions of interest for each sample.

Figure 5 displays the peak wavelengths and FWHM (full width at half maximum) analyzed from spectral profiles collected from a wide range of samples including AuNPs alone, AgNPs alone, GUVs alone, GUV+AuNPs, and GUV+AgNPs, noting that GUVs used were of varied composition including DMPC alone and 10, 20, 30, 40 mol% of CHOL:DMPC. These two graphs are the most comprehensive exhibition of the experiments conducted in this study. Quite a few data points here have been shown in Figure 1, Figure 2, Figure 3 and Figure 4 as spectral profiles. Each data point in the graph is the mean and the standard deviation calculated from the spectral profiles of 15 regions of interest selected from that sample.

It is found in Figure 5a and our data chart that the mean peak wavelength of AuNPs is 591 nm and the mean peak wavelength of AgNPs is 548 nm. The peak wavelengths for GUVs of varied composition are around 528 nm except for GUVs of 20 mol% CHOL:DMPC which peaks at 549 nm, coinciding with the peak wavelength of AgNPs. The red shifts of peak wavelength are observed for all cases of GUV+AuNPs and GUV+AgNPs from those of NPs alone. The peak wavelength of GUV+AuNPs shifts to ~616 nm from 591 nm for AuNPs alone, an increase of 25 nm. The peak wavelength of GUV+AgNPs shifts to ~579 nm from 548 nm for AgNPs alone, an increase of 31 nm. In general, the peak wavelengths AuNPs and AgNPs are both of small errors, as expected from the uniformity of particles in size and shape. However, even smaller errors are found for GUVs alone at 40 mol% CHOL:DMPC and GUV+AuNPs at 30 and 40 mol% CHOL:DMPC, suggesting a very stable peak wavelength for high concentration of CHOL especially when interacting with AuNPs. In contrast, much greater errors are noticed for peak wavelengths of GUV+AgNPs for all different compositions of GUVs, indicating that the peak wavelengths shift widely when GUVs interact with AgNPs. This stark contrast in peak wavelength shifts between AuNPs and AgNPs upon interacting with GUVs may suggest a fundamental difference of the underlying mechanism governing the NP-membrane interaction. Also worth noting is the GUV composition of 20 mol% CHOL:DMPC, which not only sees the greatest peak wavelength among all GUV compositions but also the greatest errors for GUVs only and GUV+AgNPs.

It is found in Figure 5b and our data chart that the mean FWHM of AuNPs is 151 nm and the mean FWHM of AgNPs is 111 nm. The FWHM for GUVs of varied composition are around 210 nm except for GUVs of 20 mol% CHOL:DMPC whose FWHM is 235 nm. The broadening of FWHM is noted for all cases of GUV+AuNPs and GUV+AgNPs from those of NPs alone. The FWHM of GUV+AuNPs increases to ~186 nm from 151 nm for AuNPs alone, a broadening of 35 nm. The FWHM of GUV+AgNPs increases to ~226 nm from 111 nm for AgNPs alone, an astounding broadening of 115 nm, more than doubled. This stark contrast in FWHM broadening between AuNPs and AgNPs upon interacting with GUVs further suggests a difference underlying NP-membrane interactions. Again, at GUV composition of 20 mol% CHOL:DMPC, the greatest error is found for GUV+AgNPs as is the case for peak wavelength of GUV+AgNPs at this GUV composition. GUV+AgNPs at GUV composition of 20 mol% CHOL:DMPC is the most interesting case among all plotted as it shows the greatest error for both the peak wavelength and the FWHM.

Another noticeable general trend observed in Figure 5b is that the mean FWHM of GUVs of all different compositions is ~215 nm, 29 nm broader than that of GUV+AuNPs with FWHM at ~186 nm whereas 11 nm narrower than that of GUV+AgNPs with FWHM at ~226 nm. It is somewhat counterintuitive that AuNPs decrease the spectral width of GUVs upon NP-membrane interactions whereas AgNPs increase the spectral width of GUVs upon NP-membrane interactions.

### 2.3. DFM Images of GUVs of Varied Composition Interacting with AuNPs or AgNPs

**Figure 6:**
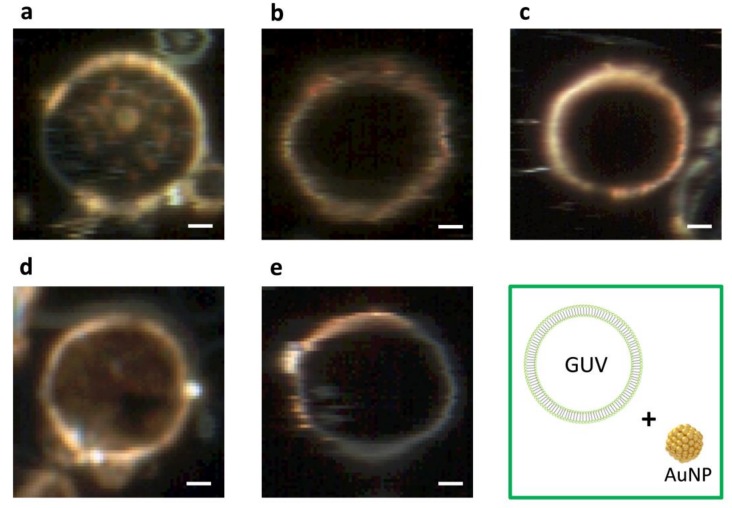
DFM images of GUV+AuNPs where GUVs are of varied composition including (**a**) DMPC only, (**b**) 10 mol%, (**c**) 20 mol%, (**d**) 30 mol%, and (**e**) 40 mol% CHOL:DMPC, respectively. Scale bars are 5 μm.

**Figure 7:**
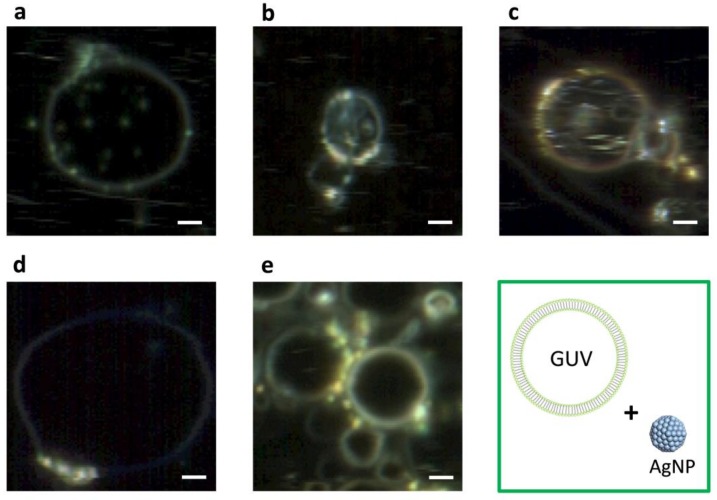
DFM images of GUV+AgNPs where GUVs are of varied composition including (**a**) DMPC only, (**b**) 10 mol%, (**c**) 20 mol%, (**d**) 30 mol%, and (**e**) 40 mol% CHOL:DMPC, respectively. Scale bars are 5 μm.

Figure 6 displays an array of DFM images of GUV+AuNPs and Figure 7 displays an array of DFM images of GUV+AgNPs, where GUVs were made of varied composition including DMPC alone and 10, 20, 30, or 40 mol% CHOL:DMPC. When AuNPs interact with GUVs, they tend to form a continuous coverage on the vesicle, forming a golden-brown crust with rarely distinguishable isolated NPs (Figure 6). On the other hand, when AgNPs interact with GUVs, they tend to anchor on the lipid membranes bejeweled on the vesicle surface with much distinguishable isolated NPs (Figure 7).

It is also noticed that among all images displayed, GUVs made of DMPC alone are most permeable, allowing the translocation of noticeable amount of AuNPs or AgNPs inside the vesicles (Figure 6a and Figure 7a). However, AuNPs found inside the vesicles (Figure 6a) are fuzzy, bulky and agglomerated whereas AgNPs found inside the vesicles (Figure 7a) are well-defined, isolated and dispersed. This suggest that AuNPs are more integrated in the lipid bilayers than AgNPs, therefore upon entry into the vesicles lipid coating was carried with AuNPs but rarely with AgNPs. By comparing the NPs inside the vesicles in the image array of Figure 6 and Figure 7, it can be found that the permeability of GUVs in general decreases with the increasing molar concentration of CHOL. This makes sense because the addition of CHOL in GUVs modulates the membrane phase and stiffens the bilayer. AgNPs are more capable of penetrating across the lipid membrane than AuNPs, as AgNPs are still spotted inside GUVs when the concentration of CHOL increased to 10 and 20 mol% (Figure 7b,c). However, the leakage is not observed for AuNPs inside CHOL-doped GUVs (Figure 6b–e).

## 3. Discussion

The distinct spectral shape and peak wavelength as seen for the spectral profiles from AuNPs vs. AgNPs (Figure 1b vs. Figure 2b) demonstrate that the hyperspectral function of DFM is capable of characterizing NPs regarding their types, shapes, and sizes. The spectral profiles as a result of scattering and reflectance from plasmonic NPs are sensitive to the dielectric medium in the surrounding, thus can be further characterized upon adsorption to lipid membranes to understand the interactions. Red shifts of 25 nm and 31 nm are observed for AuNPs and AgNPs respectively, upon interacting with GUVs (Figure 5). Previously, large red shifts were observed for AuNPs as attributed to NP cluster formation [28]. Shifts in the optical spectra of AgNPs are also expected due to changes in the surrounding dielectric medium upon adsorption, but such shifts are often much lower (~5–10 nm) [29]. Surface plasmons are charge density oscillations confined to the surface of the metal NP. When NPs form cluster, the plasmons undergo hybridization due to interparticle interactions [30]. When the interparticle distance between two NPs is within the range of Coulomb interaction, the charge density oscillations from two individual NPs hybridize to form renormalized plasmon energies. We attribute the red shifts of AuNPs and AgNPs upon interactions with GUVs to a coupled effect of NP aggregation and the surface attachment of lipid molecules.

The results from DFM imaging reveal the morphological difference between GUV+AuNPs and GUV+AgNPs (Figure 6 and Figure 7). AuNPs tend to form a continuous golden-brown crust on the membrane surface whereas AgNPs are bejeweled on the membrane as isolated particles or clusters. This difference of NPs in membrane attachment or integration as observed in spatial imaging is also reflected in the significant difference between the spectral linewidth from GUV+AuNPs and GUV+AgNPs. We learned from spectral analysis of all samples in Figure 5 that AuNPs tend to decrease the FWHM of GUVs whereas AgNPs tend to increase the FWHM of GUVs. Overall, GUV+AgNPs have much greater errors than GUV+AuNPs for both peak wavelengths and FWHM. Among the many causes for optical spectral broadening, we find that the inhomogeneous broadening is the most likely cause for the broadening of GUV+AgNPs. The morphological observation does confirm that GUV+AgNPs adopt much varied configurations than GUV+AuNPs do. The surfaces, grain boundaries, and stoichiometry variations are more pronounced in GUV+AgNPs than in GUV+AuNPs. Therefore, the emitting particles in GUV+AgNPs in much diverse local environments would emit at much different frequencies than particles from GUV+AuNPs do, causing a broadening. On the other hand, because of the uniformity of GUV-AuNP interactions, the spectral linewidths are even smaller than those from GUVs alone.

Based on the differences in morphology and spectral responses between GUV+AuNPs and GUV+AgNPs, we hypothesize that there exists a difference in the mechanism of interactions between AuNPs/AgNPs and GUVs. AuNPs are more likely integrated in the lipid bilayer. If AuNPs were simply adsorbed on the lipid bilayer (or co-localization physically), the resulting spectral profile should have been the overlap of those of AuNPs and GUVs alone. Since the average peaks of GUVs or AuNPs alone were ~580 nm and ~590 nm respectively, the expected spectral peak of the overlap should be between 580 and 590 nm. However, the average spectral peak of GUV+AuNPs is ~615 nm, longer than either alone. This may likely be the result of dampened surface plasmonic effect on AuNPs when they are trapped in between the lipid bilayer. When it comes to AgNPs, they mostly get adsorbed on the lipid bilayer as individual NPs or NP clusters. The spectral peaks of the GUV+AuNPs have large variations shifting between 540 nm and 635 nm with respect to ~550 nm for AgNPs alone. The interactions between AgNPs and GUVs are more varied than between AuNPs and GUVs. Integration and adsorption are equally likely for AgNP-GUV interaction, while integration is more prevalent for AuNP-GUV interaction.

**Figure 8:**
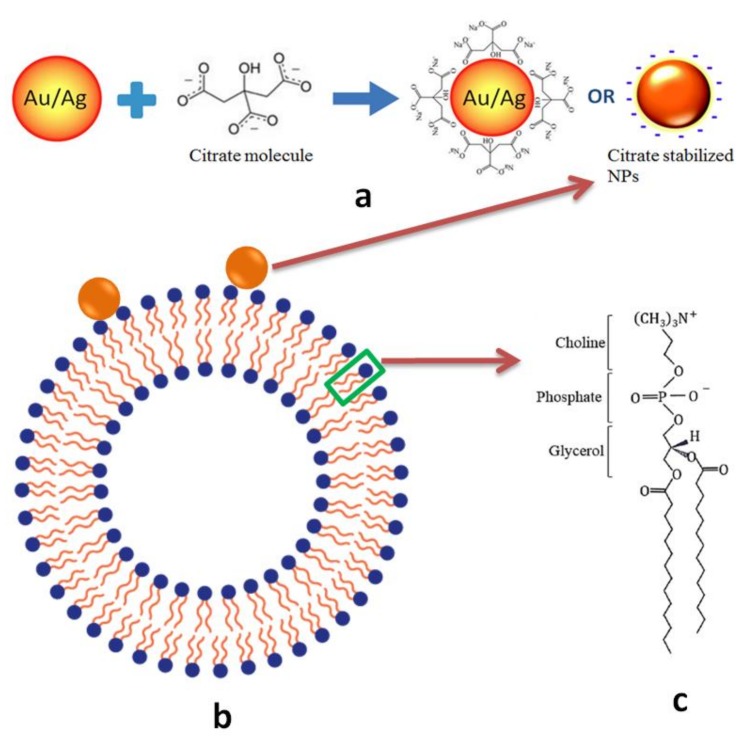
(**a**) Schematic of citrate stabilized NPs. (**b**) Schematic of a giant unilamellar vesicle (GUV) attached with a nanoparticle. (**c**) The molecular structure of DMPC.

The attachment of AuNPs and AgNPs on GUV surfaces is an inevitable first step between NP-membrane interaction because of the electrostatic force between the negatively charged citrate layer around the NPs (Figure 8a) and the cationic amine and anionic phosphate groups in the outer layer of GUVs (Figure 8b,c). The electrostatic interactions are not only essential for NP adhesion, but also critical for disruption of membranes [31]. Despite that Au and Ag are in the same group on the periodic table, Au has a higher effective nuclear charge relative to Ag. For this reason, citrate anions in the particle solvation sphere should bind more tightly to AuNPs than to AgNPs. Therefore, we hypothesize that the ability of cations to disrupt the outer layer of lipid membranes occurs at a much greater extent for AuNPs than for AgNPs. Numerous experimental and simulation studies have reported the disruption of lipid bilayers by AuNPs [27,31,32,33,34]. In the mean while when the AuNPs disrupt the outer leaflet of the lipid bilayer, the citrate anions are likely stripped off and left in the aqueous solution as the hydrophobic force between the acyl chains of DMPC and AuNPs outweighs the solvation force. We hypothesize that AuNPs trapped in the hydrophobic bilayer attempt to relieve its excess of surface energy by recruiting more AuNPs from solution (initially assisted by the disruptive electrostatic force) and seeding the growth of crust in the bilayer [32]. Once AuNPs are integrated in the lipid bilayer, the hydrophobic interaction between AuNPs and lipid chains as well as the Van der Waals (VDW) between AuNPs maintain the stability and equilibrium of the formation. No crust formation was observed for AgNPs, because they are not disruptive enough to initiate the entrapment in the bilayer.

GUVs of composition at 20 mol% CHOL:DMPC were identified to exhibit distinct spectral properties as compared to the others. This concentration coincides with the fact that typical nucleated mammalian cell contains between 10% and 20% of cholesterol out of total lipids [35]. Cholesterol plays an important role when it comes to controlling the size and area fraction of lipid phase domains in membranes. In the absence of cholesterol, phospholipid bilayers exist in either a highly ordered gel phase or a liquid-disordered (*l*_d_) phase depending on the temperature. When cholesterol is in presence, an intermediate phase called liquid-ordered (*l*_o_) is formed. Liquid-ordered lipid domains provide anchorage platforms for membrane proteins to form lipid rafts, which are floating microdomains on cell membrane for protein trafficking and cell signal transduction [17]. In previous phase studies of lipid mixture using fluorescence recovery after photobleaching (FRAP), it was found that the percolation threshold concentration of cholesterol is 20–25 mol% in lipid mixture [36]. Percolation threshold is the point where rafts become connected and fluid domains disconnected, when 45–50% of the total membrane is converted to the *l*_o_ phase. Beyond 20–25 mol%, cholesterol causes the size of the lipid rafts to decrease [37]. This critical concentration of cholesterol at 20 mol% in a lipid mixture is well coincided in our reflectance spectral analysis of GUVs (Figure 5). At 20 mol% CHOL:DMPC, the peak wavelength of GUVs has a redshift of ~20 nm as compared to other composition and the linewidth is ~25 nm broader than the rest. The 20 mol% CHOL in DMPC seems to be an ideal and economical concentration for GUVs to achieve the best integrity and the least permeability because the liquid-ordered domains have the largest fraction in total membrane area as compared to the other CHOL concentrations. This consistence with other studies also suggests that the hyperspectral analysis technique can be used to characterize the phase and integrity of lipid membranes.

## 4. Materials and Methods

### 4.1. Colloidal AuNPs and AgNPs

The AuNPs (10 nm, 0.06 mg/mL, Sigma-Aldrich #752584, sourced from CytoDiagnostics, Inc. Burlington, ON, Canada) used in this work were prepared by reduction of chloroauric acid (HAuCl4) and citrate-stabilized in 0.1 mM PBS [38]. The AgNPs (10 nm, 0.02 mg/mL, Sigma-Aldrich #730785) used in this work were prepared by the reduction of silver nitrate and citrate-stabilized in aqueous buffer [39]. The size of 10 nm was chosen in this study because our earlier work showed that 10-nm AuNPs were most effective in inducing the phase and shape changes in lipid vesicles [27]. The TEM images of AuNPs and AgNPs (Figures S1a and S2) used in this study and more details on size distribution (Figure S1b), concentration and citrate stabilization (Figure S3) can be found in the Supplementary Material.

### 4.2. Electroformation of GUVs at Various Molar Concentration of Cholesterol vs. DMPC

Electroformation of GUVs is a lab preparation technique for reproducible and controllable production of giant liposomes [40]. It involves the application of an external electric field onto lipid films soaked in hydrating solvent to induce swelling and subsequent vesicle formation. In an improved approach, the alternating (AC) instead of direct (DC) electric field was applied to introduce constant changes in both direction and magnitude of the field intensity, thus agitate lipid molecules to self-assemble into unilamellar bilayer packing and to bud into spherical structures [41]. The AC electroformation of GUVs has enabled various studies on tuning lipid compositions, domain formation and membrane mechanical properties.

The Vesicle Prep Pro apparatus (Nanion Technologies, Munich, Germany, Figure S4) was used for the electroformation of GUVs. A stock solution of phospholipid DMPC (1,2-dimyristoyl-sn-glycero-3-phosphocholine, Sigma-Aldrich) in chloroform (CHCl_3_) at 6 mg/mL was first prepared. In the case of GUVs at various molar concentration of CHOL to DMPC, a stock solution of CHOL/CHCl_3_ at 10 mg/mL was mixed into the stock solution of DMPC/CHCl_3_ at 10, 20, 30, 40 molar percent (mol%) of CHOL:DMPC. Approximately 20 µL of stock solution was dropped on the conducting side of an ITO-coated slide followed by the vacuum evaporation of solvent. A greased O-ring (diameter 28 mm) was then placed around the dried film and filled with 500 µL sorbitol (210 mM). A second ITO slide with conductive side facing down was placed on top of the O-ring to sandwich the soaked film. The ITO slide set was thereafter fit in the electrode chamber of the Vesicle Prep Pro apparatus (Figure S5). Then an alternating voltage of 5 V (p-p) at 5 Hz was applied to the slide chamber at 36 °C. After two hours of running the AC voltage, GUVs are formed and harvested in a vial for future use. More information on the GUV fabrication including the picture of the apparatus and the illustration ITO chamber can be found in Supplementary Material.

### 4.3. Hyperspectral Dark-Field Microscopy Imaging

20 µL of GUVs of varied composition (DMPC alone and DMPC doped with 10, 20, 30 or 40 mol% of CHOL) were incubated with or without 5 µL of AuNPs or AgNPs (10 nm in size) for two hours prior to the microscopy inspection. To prepare for a sample slide, a drop of 0.5 µL of incubated mixture was streaked on the slide and a cover slip was carefully flapped on the sample to minimize bubble formation. The sample slide was then mounted on the dark-field microscope (CytoViva, Inc., Auburn, AL, USA) for both spatial and hyperspectral imaging. The hyperspectral images were collected and analyzed with ENVI (ENvironment for Visualizing Images, Version 4.8, Harris Geospatial, Boulder, CO, USA), a software application originally designed for the process and analysis of geospatial imagery.

### 4.4. Analysis of Peak Wavelength and FWHM

The peak wavelength and FWHM (full width at half maximum) were determined from spectral profiles collected from a wide range of samples including AuNPs alone, AgNPs alone, GUVs alone, GUV+AuNPs, and GUV+AgNPs. GUVs tested were of varied composition ranging from DMPC alone to 10, 20, 30, 40 mol% of CHOL:DMPC. For each sample analysis, approximately 15 different regions were selected from several hyperspectral DFM images. In each region of interest, approximately 10 points were selected to produce the average spectral profile. The peak wavelength and FWHM were then determined from the exported spectral data by finding the wavelength corresponding to the maximum intensity and spectral width at half of the maximum intensity. The determination of peak wavelength and FWHM was performed for all 15 regions of interest for each sample. Then the data of peak wavelength and FWHM were averaged to produce the mean and the standard deviation for each sample.

## 5. Conclusions

Herein, we present the imaging and spectral results obtained from hyperspectral dark-field microscopy to show the interactions between AuNPs/AgNPs and GUVs. We found that citrate stabilized colloidal AuNPs or AgNPs of 10 nm interact with GUVs very differently. AuNPs tend to integrate in between the lipid bilayer and form a uniform golden-brown crust on vesicles, whereas AgNPs are bejeweled on the vesicle surface as isolated particles or clusters with much varied configurations. The greater ability of AuNPs in disrupting lipid membrane than AgNPs is hypothesized to underlie the different ways AuNPs and AgNPs interact with GUVs. The membrane disruptive ability of AuNPs allows them to entrap in between bilayers and aggregate with other AuNPs to form a golden-brown crust. The permeability of GUVs in general decreases with the increasing molar concentration of CHOL as expected. Among various compositions of GUVs, 20 mol% CHOL:DMPC was found to be an ideal concentration for GUVs to achieve the best integrity and the least permeability, consistent with the finding of other phase studies of lipid mixture that the liquid-ordered domains have the largest area fraction of the entire membrane at 20 mol% of cholesterol. Thereby, hyperspectral analysis is suggested to be a possible technique for phase and integrity characterization of lipid membranes. Upon penetration inside the vesicles at low CHOL concentration, AuNPs were more likely coated with lipid molecules than AgNPs. These results and findings have helped better understand the mechanisms of AuNP/AgNP and membrane interactions, which are fundamental and critical for their future applications in cancer nanotechnology.

## Supplementary Materials

Supplementary materials can be found at http://www.mdpi.com/1422-0067/19/4/1014/s1.

## Abbreviations

AuNPs	Gold nanoparticles
AgNPs	Silver nanoparticles
CHOL	Cholesterol
DFM	Dark-field microscopy
DMPC	Dimyristoyl phosphatidylcholine
FWHM	Full width at half maximum
GUVs	Giant unilamellar vesicles
HSI	Hyperspectral imaging
SWIR	Short wave infrared
VNIR	Visible near infrared

## References

[1] Connor E., Mwamuka J., Gole A., Murphy C., Wyatt M. (2005). Gold Nanoparticles Are Taken Up by Human Cells but Do Not Cause Acute Cytotoxicity. Small.

[2] Bhattacharya R., Mukherjee P. (2008). Biological properties of “naked” metal nanoparticles. Adv. Drug Deliv. Rev..

[3] Dykman L., Khlebtsov N. (2012). Gold nanoparticles in biomedical applications: Recent advances and perspectives. Chem. Soc. Rev..

[4] Tiwari P.M., Vig K., Dennis V.A., Singh S.R. (2011). Functionalized gold nanoparticles and their biomedical applications. Nanomaterials.

[5] Papasani M.R., Wang G., Hill R.A. (2012). Gold nanoparticles: The importance of physiological principles to devise strategies for targeted drug delivery. Nanomed. Nanotechnol. Biol. Med..

[6] Mieszawska A.J., Mulder W.J.M., Fayad Z.A., Cormode D.P. (2013). Multifunctional Gold Nanoparticles for Diagnosis and Therapy of Disease. Mol. Pharm..

[7] Chuang Y.-C., Li J.-C., Chen S.-H., Liu T.-Y., Kuo C.-H., Huang W.-T., Lin C.-S. (2010). An optical biosensing platform for proteinase activity using gold nanoparticles. Biomaterials.

[8] Wang S., Chen K.-J., Wu T.-H., Wang H., Lin W.-Y., Ohashi M., Chiou P.-Y., Tseng H.-R. (2010). Photothermal Effects of Supramolecularly Assembled Gold Nanoparticles for the Targeted Treatment of Cancer Cells. Angew. Chem. Int. Ed..

[9] Lee K., Lee H., Bae K.H., Park T.G. (2010). Heparin immobilized gold nanoparticles for targeted detection and apoptotic death of metastatic cancer cells. Biomaterials.

[10] Abdelgawad A.M., Hudson S.M., Rojas O.J. (2014). Antimicrobial wound dressing nanofiber mats from multicomponent (chitosan/silver-NPs/polyvinyl alcohol) systems. Carbohydr. Polym..

[11] Arvizo R.R., Bhattacharyya S., Kudgus R.A., Giri K., Bhattacharya R., Mukherjee P. (2012). Intrinsic therapeutic applications of noble metal nanoparticles: Past, present and future. Chem. Soc. Rev..

[12] Jain S., Hirst D.G., O’Sullivan J.M. (2012). Gold nanoparticles as novel agents for cancer therapy. Br. J. Radiol..

[13] Jeyaraj M., Sathishkumar G., Sivanandhan G., MubarakAli D., Rajesh M., Arun R., Kapildev G., Manickavasagam M., Thajuddin N., Premkumar K. (2013). Biogenic silver nanoparticles for cancer treatment: An experimental report. Colloids Surf. B.

[14] Mu Q., Jiang G., Chen L., Zhou H., Fourches D., Tropsha A., Yan B. (2014). Chemical Basis of Interactions Between Engineered Nanoparticles and Biological Systems. Chem. Rev..

[15] Fenz S.F., Sengupta K. (2012). Giant vesicles as cell models. Integr. Biol..

[16] Simons K., Ikonen E. (1997). Functional rafts in cell membranes. Nature.

[17] Pike L.J. (2003). Lipid rafts: Bringing order to chaos. J. Lipid Res..

[18] Resh M.D. (1999). Fatty acylation of proteins: New insights into membrane targeting of myristoylated and palmitoylated proteins. Biochim. Biophys. Acta.

[19] Hanahan D., Weinberg R. (2011). Hallmarks of Cancer: The Next Generation. Cell.

[20] Aruffo A., Stamenkovic I., Melnick M., Underhill C.B., Seed B. (1990). CD44 is the principal cell surface receptor for hyaluronate. Cell.

[21] Thomas L., Byers H.R., Vink J., Stamenkovic I. (1992). CD44H regulates tumor cell migration on hyaluronate-coated substrate. J. Cell Biol..

[22] Günthert U., Hofmann M., Rudy W., Reber S., Zöller M., Hauβmann I., Matzku S., Wenzel A., Ponta H., Herrlich P. (1991). A new variant of glycoprotein CD44 confers metastatic potential to rat carcinoma cells. Cell.

[23] Murai T., Maruyama Y., Mio K., Nishiyama H., Suga M., Sato C. (2011). Low Cholesterol Triggers Membrane Microdomain-dependent CD44 Shedding and Suppresses Tumor Cell Migration. J. Biol. Chem..

[24] Hu M., Novo C., Funston A., Wang H., Staleva H., Zou S., Mulvaney P., Xia Y., Hartland G.V. (2008). Dark-field microscopy studies of single metal nanoparticles: Understanding the factors that influence the linewidth of the localized surface plasmon resonance. J. Mater. Chem..

[25] Roth G.A., Tahiliani S., Neu-Baker N.M., Brenner S.A. (2015). Hyperspectral microscopy as an analytical tool for nanomaterials. Wiley Interdiscip. Rev. Nanomed. Nanobiotechnol..

[26] Siddiqi A.M., Li H., Faruque F., Williams W., Lai K., Hughson M., Bigler S., Beach J., Johnson W. (2008). Use of hyperspectral imaging to distinguish normal, precancerous, and cancerous cells. Cancer Cytopathol..

[27] Bhat A., Edwards L.W., Fu X., Badman D.L., Huo S., Jin A.J., Lu Q. (2016). Effects of gold nanoparticles on lipid packing and membrane pore formation. Appl. Phys. Lett..

[28] Chen R., Choudhary P., Schurr R.N., Bhattacharya P., Brown J.M., Ke P.C. (2012). Interaction of lipid vesicle with silver nanoparticle-serum albumin protein corona. Appl. Phys. Lett..

[29] Podila R., Chen R., Ke P.C., Brown J., Rao A. (2012). Effects of surface functional groups on the formation of nanoparticle-protein corona. Appl. Phys. Lett..

[30] Nordlander P., Oubre C., Prodan E., Li K., Stockman M.I. (2004). Plasmon Hybridization in Nanoparticle Dimers. Nano Lett..

[31] Xiao X., Montaño G.A., Edwards T.L., Allen A., Achyuthan K.E., Polsky R., Wheeler D.R., Brozik S.M. (2012). Surface Charge Dependent Nanoparticle Disruption and Deposition of Lipid Bilayer Assemblies. Langmuir.

[32] Montis C., Maiolo D., Alessandri I., Bergese P., Berti D. (2014). Interaction of nanoparticles with lipid membranes: a multiscale perspective. Nanoscale.

[33] Heikkilä E., Martinez-Seara H., Gurtovenko A.A., Javanainen M., Häkkinen H., Vattulainen I., Akola J. (2014). Cationic Au nanoparticle binding with plasma membrane-like lipid bilayers: Potential mechanism for spontaneous permeation to cells revealed by atomistic simulations. J. Phys. Chem. C.

[34] Lin J., Zhang H., Chen Z., Zheng Y. (2010). Penetration of lipid membranes by gold nanoparticles: Insights into cellular uptake, cytotoxicity, and their relationship. ACS Nano.

[35] Vance J.E. (2015). Phospholipid synthesis and transport in mammalian cells. Traffic.

[36] Crane J.M., Tamm L.K. (2004). Role of cholesterol in the formation and nature of lipid rafts in planar and spherical model membranes. Biophys. J..

[37] Almeida P.F., Vaz W.L., Thompson T.E. (1993). Percolation and diffusion in three-component lipid bilayers: Effect of cholesterol on an equimolar mixture of two phosphatidylcholines. Biophys. J..

[38] Toma H.E., Zamarion V.M., Toma S.H., Araki K. (2010). The coordination chemistry at gold nanoparticles. J. Braz. Chem. Soc..

[39] Iravani S., Korbekandi H., Mirmohammadi S., Zolfaghari B. (2014). Synthesis of silver nanoparticles: Chemical, physical and biological methods. Res. Pharm. Sci..

[40] Mikelj M., Praper T., Demic R., Hodnik V., Turk T., Anderluh G. (2013). Electroformation of giant unilamellar vesicles from erythrocyte membranes under low-salt conditions. Anal. Biochem..

[41] Angelova M.I., Soléau S., Méléard P., Faucon F., Bothorel P.H.F., Helm C., Lцsche M., Mцhwald H. (1992). Preparation of giant vesicles by external AC electric fields. Kinetics and applications. Trends in Colloid and Interface Science VI.

