# Supplementary material for: Probing Interactions between AuNPs/AgNPs and Giant Unilamellar Vesicles (GUVs) Using Hyperspectral Dark-field Microscopy
Source: Int J Mol Sci. 2018 Mar 28;19(4):1014. doi: 10.3390/ijms19041014 (PMC5979378; doi:10.3390/ijms19041014)
Supplement: Supplementary file 1 [file ijms-19-01014-s001.pdf]

Probing Interactions between AuNPs/AgNPs and Giant Unilamellar Vesicles (GUVs) Using Hyperspectral Dark-field Microscopy

Supplementary Material

1. Properties of Colloidal AuNPs and AgNPs

AuNPs of 10 nm is ordered from Sigma-Aldrich (catalog# 752584). The core size of this product is 8–12 nm and the hydrodynamic diameter is 11–25 nm. AuNPs are stabilized in 0.1 mM phosphate buffered saline. The concentration of AuNPs is given as ~6.0E+12 particles/mL. Considering the atomic mass of gold being 197 and using an estimation method described in http://sustainable-nano.com/2016/07/28/how-many-atoms-are-in-a-nanoparticle/, the mass concentration of AuNPs of 10 nm is determined to be 4/3*pi*5^3/0.0679*4=30,845. The total mass of ~6.0E+12 particles is then 30845*197*6e12/6.022e23=6.05e-5 g. The mass concentration of AuNPs is then 0.06 mg/mL. The TEM image and the dynamic light scattering characterization of colloidal 10-nm AuNPs shown in Figure S1 is taken from the manufacturer's website at https://www.cytodiagnostics.com/store/pc/10nm-Reactant-Free-Gold-Nanoparticles-20ml-151p1142.htm.

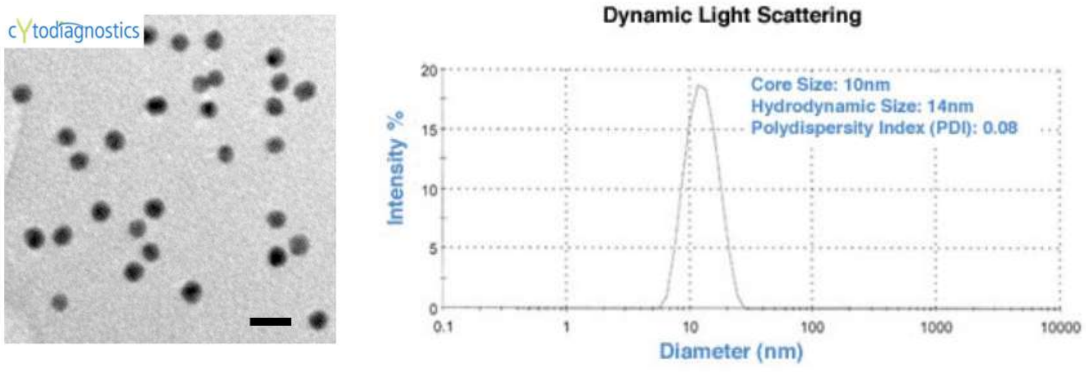

(**a**) (**b**)

Figure S1. (**a**) TEM image and (**b**) dynamic light scattering size characterization of 10-nm AuNPs. Scale bar: 20 nm.

AgNPs of 10 nm is ordered from Sigma-Aldrich (catalog# 730785). The size of this product as determined by TEM is 10 ± 4 nm. AgNPs are suspended in aqueous buffer with sodium citrate as a stabilizer. The mass concentration of AgNPs is around 0.02 mg/mL. The TEM image of colloidal AgNPs in Figure S2 is found on the product web page (https://www.sigmaaldrich.com/catalog/product/aldrich/730785).

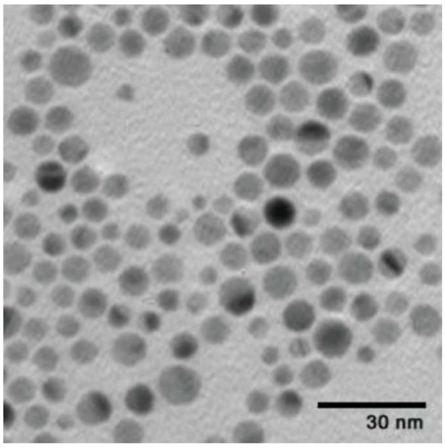

Figure S2. TEM image of 10-nm AuNPs.

The citrate stabilization of AuNPs or AgNPs is demonstrated in Figure S3.

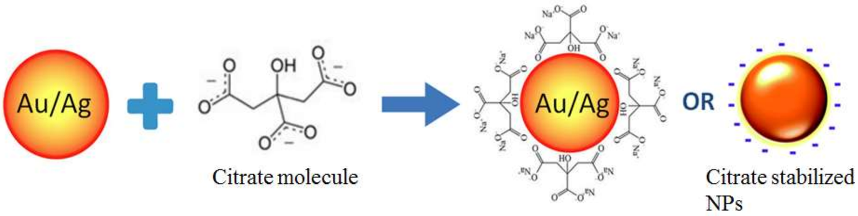

Figure S3. Schematic of citrate stabilization of AuNPs and AgNPs.

2. GUV Fabrication

Fabrication of GUV involves application of an external electric field for the process of lipid swelling and vesicle formation and this technique is called electroformation. Attempt initiated by application of static (DC) electric field was reported for the first time by Angelova and Dimitrov (1986) [1]. As found in subsequent experiments, the range of lipid types that form GUVs in DC electric field was rather narrow, perhaps because the static field exerts only an "ordering" effect on lipid molecules. This limitation was overcome by a new experimental approach in which an alternating (AC) electric field was used in GUV formation [2]. The AC field, due to the permanent (usually sinusoidal) change of both direction and magnitude of the field intensity, apart from ordering effect also forces molecules to move, what might help lipids to spontaneously adopt bilayer packing and to form unilamellar structures. This new method enabled the formation of giant vesicles using a variety of lipid mixtures and the study of different properties of their membranes. A few of the underlying mechanisms for the effects of the external electric field on the process of lipid swelling and vesicle formation are mentioned below:

- Direct electrostatic interactions between electrode and bilayers due to injection of charges from electrodes
- Electro osmotically induced mechanical stresses
- Electrochemical reactions
- Redistribution of double layer counter ions between bilayers
- Reorientation and lateral redistribution of lipid molecules

Electroformation of GUVs is the method allowing the reproducible production of giant lipid vesicles and therefore is quite often used in laboratories performing studies on model bilayers. In this method lipids solubilized in organic solvents, e.g., a mixture of chloroform and methanol, are deposited on the electrode, dried, and then exposed to an AC field in the presence of a hydrating solvent. It was proposed that mechanical stress induced by the AC field plays a role in separating and destabilizing the membranes to form giant lipid vesicles. The predominating mechanism of electroformation is the electro-osmotic periodic movement of the water medium at the water electrode interface. These vibrations are directed perpendicular to the electrode surface, where the initial lipid film is deposited, thus pulling lipid lamellae off the electrode and separating them from each other as they grow like "mushrooms". Vesicles grow in size continuously up to 20 μm. At that stage "mushrooms" start laterally connecting with each other. The zone of contact increases and at a certain moment the AC induced vibrations cause the contact zone to be destabilized, making neighboring "mushrooms" fuse together into a giant one. It takes a few minutes for the resulting "mushroom" to get spherical, close the neck and eventually separate from the electrode. The electro-osmotic vibrations are manifested as mechanical vibrations like those in the sonication of lipid/water dispersions. However, the electro-osmotic agitation is gentler, fine and can be controlled precisely. GUVs used in this researched were prepared with the Vesicle Prep Pro (Nanion Technologies, Germany) apparatus [3].

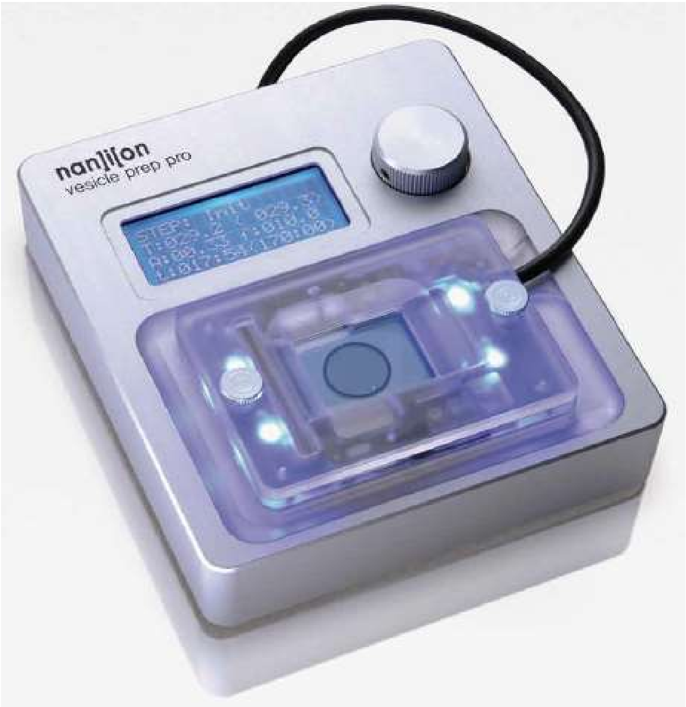

Figure S4. Vesicle Prep Pro apparatus for fabrication of GUVs [3].

The Vesicle Prep Pro (Nanion Technologies, Munich, Germany) is an automated device for preparation of GUVs ranging from 1–30 μm in diameter. They are formed by means of electro-swelling (hydration of dry lipid film in an oscillating electric field). The Vesicle Prep Pro offers a standardized and robust way to reliably and reproducibly generate GUVs of homogeneous size distribution with high yields.

The chamber used for vesicle formation consists of two glass cover slides. The slides are coated with indium tin oxide (ITO) turning them into electrodes. This leaves the chamber transparent so that vesicle formation and growth can be monitored throughout the entire process. Integrated features, including

flexible protocol design and temperature control, allow generation of GUVs from lipids with high charge or high melting temperature.

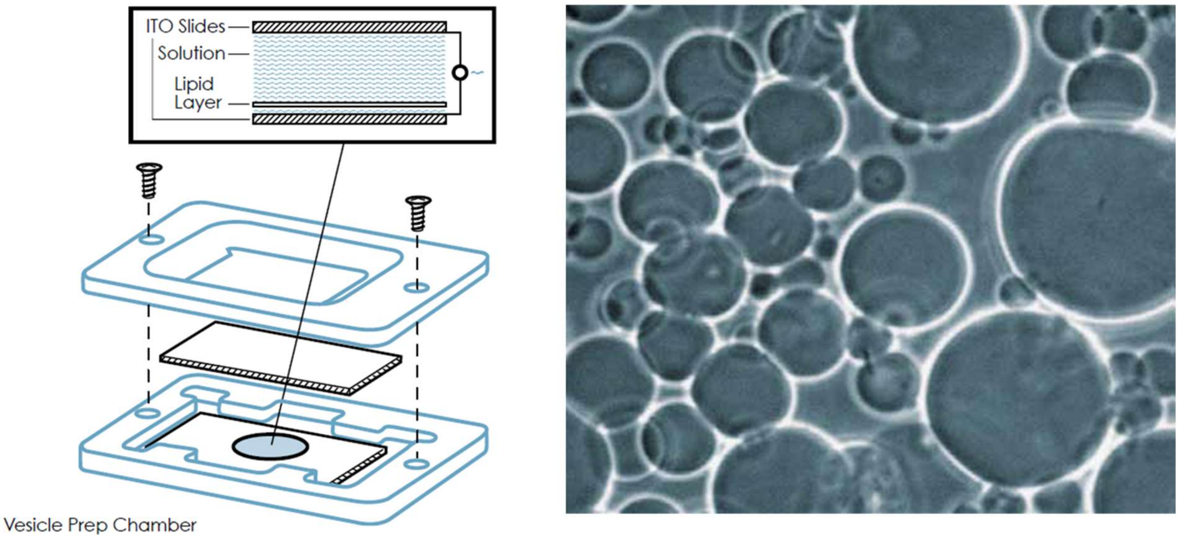

Figure S5. Diagram illustrating the setup for GUV fabrication and GUVs formed after the process [3].

Approximately 20 µL of the lipid stock solution was placed on the ITO glass surface of the Vesicle Prep Pro station and allowed to dry overnight in a vacuum chamber. Next, 18 mm O-ring was greased and placed around the dry lipid film. The lipid film was hydrated with 250 µL 0.5 M sorbitol placed inside the ring and the second ITO slide with the conducting side facing down was used to cover the O-ring. The vesicles were formed by electro-swelling under the influence of an AC electric field of 5 Vp-p amplitude and 10 Hz frequency for 2 h at 36 °C. GUVs formed were suspended and collected in a vial.

References

1. M. I. Angelova & D. S. Dimitrov, Liposome electroformation, Faraday Discuss. Chem. Soc., (1986), 303–311.
2. M. I. Angelova, S. Soléau, P. Méléard, F. Faucon & P. Bothorel, Preparation of giant vesicles by external AC electric fields. Kinetics and applications, Progress in Colloid & Polymer Science, (1992), 127–131.
3. Vesicle Prep Pro: First product on the market for automated preparation of solvent free giant unilamellar vesicles, [Brochure], Nanion Technologies.
